# Acute esophageal necrosis following cardiac arrest: A rare and lethal syndrome with diagnostic challenges

**DOI:** 10.1016/j.ijscr.2024.109751

**Published:** 2024-05-10

**Authors:** Erik Roman-Pognuz, Sara Rigutti, Giulia Colussi, Enrico Lena, Marco Bonsano, Umberto Lucangelo

**Affiliations:** a Intensive Care Unit, University Hospital of Cattinara - ASUGI, Trieste, Strada di Fiume 445, 34100 Trieste, Italy; b Department of medical science, University of Trieste, Strada di Fiume 445, 34100 Trieste, Italy

**Keywords:** Acute esophageal necrosis (AEN), Upper gastrointestinal bleeding, Esophageal ischemia, Cardiac arrest, Resuscitation, Case report

## Abstract

**Introduction and clinical relevance:**

Acute esophageal necrosis (AEN) is a condition characterized by the necrosis of the distal portion of the esophageal mucosa. Risk factors predisposing to this condition are associated to compromised vascular perfusion (*e.g.* diabetes mellitus, chronic kidney disease, advanced age, and hypertension, shock states). Complications of AEN can be severe including UGI stricture, perforation and overall increased mortality. The true incidence of AEN remains uncertain due to potential subclincal presentations and early resolution.

**Case presentation:**

The case outlined involves a 66-years-old obese male with history of alcoholism and lymph-edema of the left leg who presented to the emergency department with hematemesis, haemodynamic instability and impaired consciousness. Shortly after initial assessment, the patient went into cardiac arrest with pulse-less electrical activity (PEA). Return of spontaneous circulation (ROSC) was achieved following instigation of ALS protocol, fluid resuscitation and the administration of a total of 5 mg of adrenaline. Following stabilization, a CT scan was performed which reported a moderately enlarged esophagus with a thickened wall, liquid hypodense material within the esophagus and stomach, and liver cirrhosis. The emergent esophagogastroduodenoscopy (EGDS) revealed extensive mucosal findings indicative of diffuse necrosis with initial scarring, which was later diagnosed as AEN. The patient unfortunately deceased in ICU after developing progression of the AEN, post-cardiac arrest syndrome and liver failure.

**Clinical discussion:**

The presented case highlights several crucial clinical issues and management problems related to AEN. To diagnose AEN, EGDS is still the gold-standard since it allows direct inspection of the esophageal mucosal layer. The management of AEN necessitates a multidisciplinary approach that includes aggressive resuscitation, treatment of underlying comorbidities, and supportive care (*e.g.* proton pump inhibitors). The mortality rate for AEN remains high despite improvements in diagnosis and treatment highlighting the need to recognize this condition early and intervene promptly in the patients affected. Moreover, long-term sequelae like stricture formation of the esophagus and impaired esophageal motility may contribute to morbidity requiring continuos monitoring. Therefore, to optimize outcomes while reducing complications among affected patients, prompt identification associated with appropriate medical measures are essential. More research needs to be done aiming to better understand the pathophysiology of AEN thereby identifying strategies for its prevention or cure.

**Conclusions:**

AEN is a rare syndrome characterized by upper gastrointestinal bleeding and hypoxic damage of the esophageal mucosa, often associated with ischemia, gastric outlet obstruction, and compromised protective barriers. Treatment involves aggressive resuscitation, proton pump inhibitors, and monitoring for infection or perforation. However, despite intensive efforts, the mortality rate for AEN remains high at 32 %.

## Highlights

• Critical Patient: 66-year-old obese male with possible alcoholic liver disease and recent left leg lymphedema. Presented with coffee ground emesis and signs of hypoperfusion.
• Endoscopy & Treatment: Emergent upper GI endoscopy revealed pale, granular esophageal mucosa with black membranes, indicating diffuse necrosis with initial scarring. Treated with pantoprazole, vasopressors, transfusions, and renal therapy due to liver failure and coagulation deficit. Follow-up EGD showed advanced scarring.
• Implications: Acute Esophageal Necrosis (AEN) links to ischemia, gastric obstruction, and compromised barriers. Diagnosis through endoscopy is crucial due to CT's limited specificity. Despite aggressive treatment, mortality rates are high, tied to underlying conditions.
• Novel Research: This study sheds light on AEN's links to cardiac arrest, ischemia, and barriers. It introduces innovative diagnostic methods, particularly emergent upper GI endoscopy, revealing distinct mucosal changes of necrosis with initial scarring. Advancing understanding in cardiac arrest scenarios and diagnostic approaches for this rare syndrome is crucial.

## 1 Introduction

Acute esophageal necrosis (AEN), also referred to as “black esophagus” or “acute necrotizing esophagitis,” is a rare and life-threatening clinical condition characterized by a peculiar endoscopic appearance at the esophagogastroduodenoscopy (EGDS). In AEN, the distal esophageal mucosa displays a circumferential black discolouration, with a characteristic sharp demarcation between necrotic and healthy tissue at the level of gastroeophageal junction (GEJ) [[1], [2], [3]]. The distal esophagus is typically affected in 97 % of cases, reflecting the relatively sparse distribution of vascular supply in this region [[4], [5], [6], [7]].

The pathogenesis and etiology of AEN may involve multiple factors, such as backflow of gastric contents causing direct esophageal injury, disruption of the vascular supply leading to hypoperfusion and ischemia, and impaired protective barrier systems due to a hemodynamically unstable state [8,9].

Individuals at risk of developing low-flow sates in the context of compromised vascular perfusion are at higher risk of reporting ischemic esophageal injury and, subsequently, AEN. Predisposing factors include diabetes mellitus, renal impairment, advanced age, and hypertension. Moreover, patients with fragility, such as those with malignancy, chronic alcoholism, chronic liver disease, or chronic kidney disease, may have a weakened protective barrier system that is less effective in shielding the esophageal mucosa from direct injuries. These patients may also have impaired regenerative capabilities of the esophageal epithelium, making it more susceptible to damage [10,11].

The most common initial presentation of patients with AEN are sysmptomps consitent with upper GI bleeding, such as haemathemesis and melena, that are present in up to 80 % of the cases, associated with haemodynamic instability [2,9]*.* Therefore, the differential diagnosis should consider other conditions that might present with symptomps suggesive of upper gastrointestinal bleeding, such as: malignant melanoma, melanocytosis, pseudomelanosis, coal dust deposition, acanthosis nigricans, and caustic ingestion [2].

While endoscopic appearance is typically diagnostic, histological correlation can be helpful in confirming the diagnosis and ruling out associated infectious conditions such as candidiasis, herpes simplex virus, or cytomegalovirus [3,4].

Complications of AEN may include stenosis or stricture formation in the distal esophagus, perforation, mediastinitis, and in severe cases, death [2].

Large retrospective series have estimated the incidence of AEN among patients undergoing EGDS at approximately 0.2–0.28 % [7,13]*.* However, there is a high suspicion that the condition may be underdiagnosed [12]*.* This condition can present with subclinical symptoms or the mucosa might heal early after transient ischemic or chemical injury [2], as supported by autopsy studies that have reported esophageal necrosis in up to 10.3 % of cases [[14], [15], [16]]*.*

This case report endeavors to provide a comprehensive and detailed analysis of Acute Esophageal Necrosis (AEN), delving into its underlying causes, mechanisms, diagnostic approaches, management strategies, and potential complications.

## 2 Case presentation

A 66-year-old obese male, with unconfirmed history of alcoholic liver disease and recent lymphedema of the left leg, presented to the emergency department with hematemesis, haemodynamic instability and impaired consciousness.

**Fig. 1:**
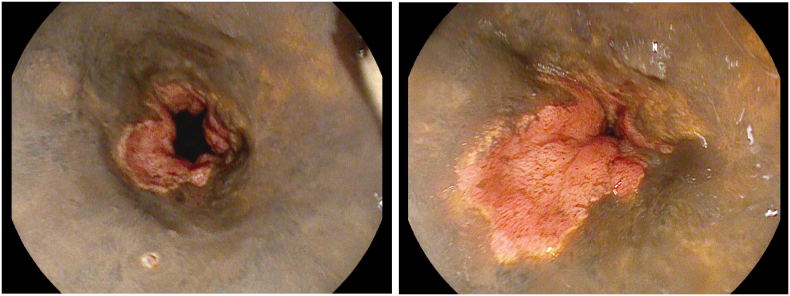
a: Pale and granular esophageal mucosa with distal black membranes of diffuse necrosis with initial scarring. b: Granular esophageal mucosa covered with delicate granulation tissue with an advanced phase of scarring.

The patient was initially evaluated by the Medical Emergency Team and found to be lethargic, tachypneic, and hypotensive, ultimately progressing to cardiac arrest with an initial rhythm of pulseless electrical activity (PEA). Prompt instigation of Advanced Life Support (ALS) led to Return of Spontaneous Circulation (ROSC) after 15 min of cardio-pulmonary resuscitation, requiring the administration of a total of 5 mg of adrenaline. Inhalation of gastric contents was evident during intubation. Further diagnostic workup, including a CT scan, revealed a moderately enlarged esophagus with a thickened wall, along with liquid hypodense material in the esophagus and stomach. Liver cirrhosis with diffuse collateral vessels was also identified. Upon admission to the Intensive Care Unit (ICU), the patient was found to be afebrile, tachycardic, and with severe hypotension necessitating high dose vasopressor support with Norepinephrine. Arterial blood gas analysis revealed severe metabolic acidosis with hyperlactatemia. An emergent EGDS was performed revealing diffuse severe mucosal changes characterized by pale and granular esophageal mucosa with distal black membranes, suggestive of diffuse necrosis with initial scarring (Fig. 1 a, b). The gastric mucosa and duodenal bulb appeared hyperemic with scattered small erosions.

The patient was treated with high doses of intravenous pantoprazole. The insertion of a nasogastric tube was avoided to prevent further damage to the ischemic mucosa. Therefore, total parenteral nutrition was initiated.

Ongoing aggressive resuscitation was required, including high-dosage vasopressors, hemocomponent transfusions, and renal replacement therapy due to evolving liver failure with coagulation factor deficit. Empirical broad-spectrum antibiotic therapy was also initiated.

After seven days, a second EGDS revealed pale and granular esophageal mucosa covered with delicate granulation tissue, consistent with an advanced phase of scarring. The gastric mucosa was hyperemic and atrophic, with signs of scarring developing on the previously identified areas of erosion.

The patient's neurological status was poor, with a fluctuating Glasgow Coma Scale, initially at 6 (E4V1M1), and neurophysiological studies showed no abnormalities. The patient's prognosis was adversely affected by post-cardiac arrest syndrome and liver failure, and unfortunately, he succumbed to his conditions in the ICU on day 17 since admission.

Despite advancements in endoscopy leading to increased diagnosis, AEN remains a rare syndrome. Hematemesis and/or melena are commonly observed in patients, along with sepsis-associated signs such as lactic acidosis, tachycardia, and hypotension, as evident in the case being presented.

The injury pattern reported in AEN has been described by the “two hit” hypothesis. According to this theoretical model, the first insult is caused by initial systemic event (*i.e.* low-flow state), that predisposes the esophageal mucosa to a secondary direct injury (*i.e.* reflux of acid and pepsin). In addition, esophageal reflux can be increased by concomitant gastric outlet obstruction. This model is supported by the observation of extensive esophageal necrosis in patients that suffers from a temporary reduction of esophageal blood supply, that is rapidly resolved upon restoration of normal flow [18].

With regards to the case presented, co-existing conditions, such as chronic alcohol abuse, may have compromised the protective mechanisms of the esophageal mucosa, making it more susceptible to injury. The low-flow state associated with cardiac arrest and subsequent hemodynamic instability could have led to ischemia. Additionally, the acute hemorrhage observed on the chest CT that was potentially attributed to the damage to bronchial or intercostal arteries during cardiopulmonary resuscitation, might have caused further reduction in blood supply to the middle esophagus. On top of that, reflux of gastric contents into the esophagus could have caused direct injury leading to esophageal necrosis.

Given the limited specificity of thoracic CT findings, endoscopy remains the preferred and most reliable method for diagnosing AEN. The treatment approach for patients with AEN involves aggressive resuscitation and timely intervention. There is no specific medical or surgical intervention to reverse the damage; however, if detected early, AEN can be reversible.

Immediate initiation of aggressive treatment for underlying medical conditions, including hemodynamic resuscitation, glycemic control, nil-*per-os* restriction, and high-dose intravenous proton pump inhibitors for acid suppression, should be implemented in these patients [2,19]*.*

Nevertheless, despite aggressive resuscitation efforts, the mortality rate for AEN remains high at 32 % [6,7,13]*.* The clinical picture presented in this case report was consistent with the evidence described in literature. The work has been reported in line with the SCARE criteria. [20]

## 3 Conclusions

AEN is typically associated with ischemia resulting from hemodynamic compromise, gastric outlet obstruction with backflow chemical injury, and compromised protective barriers along with reduced physiological reserves due to critical illness and general deconditioning. AEN presents with signs of upper gastrointestinal bleeding and is characterized by a distinctive appearance on upper endoscopy. The esophageal mucosa appears circumferential, diffuse, black-appearing, and friable, typically affecting the medial-distal part of the organ and ending abruptly at the gastroesophageal junction. The presentation of AEN in our patient was in line with previous literature, as he exhibited epidemiological and patophysiological characteristics that have been extensively described. The management of AEN entails aggressive resuscitation, correction of underlying medical conditions, initiation of proton pump inhibitor therapy and vigilant monitoring for signs of infection or perforation. Nevertheless, the mortality rate of AEN remains elevated. Mortality from his condition is significantly affected by the severity of underlying medical conditions and the overall health status of the patient, as observed in our case.

## Ethical approval

The case report has been approved by the ethic committee with the reference number: SCRICAQARC/ASUGI 0001370 P dd. 20/12/2023.

## Funding

There is no founding sources to be declared.

## CRediT authorship contribution statement

Erik Roman-Pognuz: study concept and design, writing the paper

Umberto Lucangelo: data analysis

Marco Bonsano: data collection

Giulia Colussi: data collection

Enrico Lena: data interpretation

Sara Rigutti: data collection

## Guarantor

Erik Roman-Pognuz MD, PhD.

## Consent

Written informed consent was obtained from the next of kin for publication of this case report and accompanying images. A copy of the written consent is available for review by the Editor-in-Chief of this journal on request.

## Declaration of competing interest

No conflicts of interest were present.
